# From Anti-Severe Acute Respiratory Syndrome Coronavirus 2 Immune Response to Cancer Onset via Molecular Mimicry and Cross-Reactivity

**DOI:** 10.1055/s-0041-1735590

**Published:** 2021-09-07

**Authors:** Darja Kanduc

**Affiliations:** 1 Department of Biosciences, Biotechnologies, and Biopharmaceutics, University of Bari, Bari, Italy

**Keywords:** SARS-CoV-2 spike gp, tumor-suppressor proteins, molecular mimicry, cross-reactivity, long COVID, cancer epidemic

## Abstract

**Background and Objectives** Whether exposure to severe acute respiratory syndrome coronavirus 2 (SARS-CoV-2) may predispose to the risk of cancer in individuals with no prior cancers is a crucial question that remains unclear. To confirm/refute possible relationships between exposure to the virus and ex novo insurgence of tumors, this study analyzed molecular mimicry and the related cross-reactive potential between SARS-CoV-2 spike glycoprotein (gp) antigen and human tumor-suppressor proteins.

**Materials and Methods** Tumor-associated proteins were retrieved from UniProt database and analyzed for pentapeptide sharing with SARS-CoV-2 spike gp by using publicly available databases.

**Results** An impressively high level of molecular mimicry exists between SARS-CoV-2 spike gp and tumor-associated proteins. Numerically, 294 tumor-suppressor proteins share 308 pentapeptides with the viral antigen. Crucially, the shared peptides have a relevant immunologic potential by repeatedly occurring in experimentally validated epitopes. Such immunologic potential is of further relevancy in that most of the shared peptides are also present in infectious pathogens to which, in general, human population has already been exposed, thus indicating the possibility of immunologic imprint phenomena.

**Conclusion** This article described a vast peptide overlap between SARS-CoV-2 spike gp and tumor-suppressor proteins, and supports autoimmune cross-reactivity as a potential mechanism underlying prospective cancer insurgence following exposure to SARS-CoV-2. Clinically, the findings call for close surveillance of tumor sequelae that possibly could result from the current coronavirus pandemic.

## Introduction

From lung damages to skin diseases and excessive immune responses, the disorders associated with severe acute respiratory syndrome coronavirus 2 (SARS-CoV-2), that is, coronavirus disease 2019 (COVID-19), are progressively being defined and diagnostically cataloged. 1 2 3 4 5 6 7 Among the many diseases encompassed by COVID-19, clinical attention has focused on the relationship between SARS-CoV-2 and cancer. 8 9 10 Indeed, when compared with the pre-COVID-19 era, COVID-19 pandemic appears to be characterized by higher hospitalization and mortality rates in prostate cancer patients 11 ; increased breast cancer dimensions 12 ; increased proportion of patients with advanced non-small cell lung cancer 13 ; and a higher number of diagnosed head and neck cancers (2.9–8.06% in January–April 2020). 14 Such data have been interpreted as due to the pressure exerted by the viral pandemic on the health care system, so cancer treatments have been delayed and, also, have been related to the viral infection per se. 15 However, how SARS-CoV-2 infection might relate to cancer diseases remains unclear.

According to the research paradigm that peptide sequences common to pathogens and the human host may lead to autoimmunity through cross-reactivity, 16 17 18 19 20 21 a previous report 22 has proposed cross-reactivity as a likely mechanism that can explain the immunopathology related to SARS-CoV-2 exposure. As a matter of fact, many SARS-CoV-2-derived epitopes were shown to share peptide sequences with human proteins that are involved—when altered, mutated, deficient, and/or improperly functioning—in the etiology of the diseases encompassed by COVID-19. 22 Moreover, and of special importance, it was noted that the viral versus human peptide sharing also involved human proteins related to pleuropulmonary blastoma, non-small cell lung cancer, breast invasive ductal carcinoma, multiple human cancers, tumor predisposition syndrome, and mesothelioma, inter alia. That is, the data suggested the possibility that morbidity/mortality increases in various tumors might represent long-term sequelae following exposure to SARS-CoV-2 (Kanduc 22 and pertinent references therein).

Hence, this study was undertaken to further explore the relationship between SARS-CoV-2 infection/active immunization and carcinogenesis, and specifically focused on the amino acid (aa) sequence identities between SARS-CoV-2 spike glycoprotein (gp) and tumor-suppressor human proteins. Analyses revealed a vast peptide sharing potentially able to generate pathogenic autoantibodies via cross-reactivity and immunologic imprinting phenomena, thus possibly leading to or enhancing the onset of a wide spectrum of cancer diseases.

## Materials and Methods

Peptide sharing between SARS-CoV-2 spike gp (NCBI, GenBank Protein Accession ID = QHD43416.1) and cancer-related human proteins was analyzed using pentapeptides as sequence probes as already described. 16 17 18 19 20 21 22 Pentapeptides were used as minimal immune determinant units since a peptide grouping formed of five aa residues defines an immune unit that can (1) induce highly specific antibodies and (2) determine antigen–antibody-specific interaction (Kanduc 23 24 and further references therein). Seven hundred eighty-two human proteins (in)directly linked to cancer were obtained from UniProtKB database ( www.uniprot.org ) 25 using “tumor suppressor” as keywords and are listed by UniProt entry in Supplementary Table S1 (available in online version only).

Methodologically, the spike gp primary sequence was dissected into pentapeptides offset by one residue (i.e., MFVFL, FVFLV, VFLVL, FLVLL, and so forth) and the resulting viral pentapeptides were analyzed for occurrences within the human proteins related to cancer. The shared peptides were also controlled for occurrences in the pathogens *Bordetella pertussis* , *Corynebacterium diphtheriae* , *Clostridium tetani* , *Haemophilus influenzae* , and *Neisseria meningitides* . The publicly available peptide match and peptide search programs ( www.uniprot.org ) were used. 25

The immunologic potential of the peptides shared between SARS-CoV-2 spike gp and cancer-related proteins was investigated by searching the Immune Epitope Database (IEDB, www.iedb.org ) 26 for experimentally validated immunoreactive SARS-CoV-2 spike gp-derived epitopes hosting the shared pentapeptides.

## Results and Discussion

Searching UniProt database for tumor-suppressor proteins produced 782 protein entries (in)directly related to tumor-suppressor activity and listed in Supplementary Table S1 (available in online version only). Of the 782 proteins, 294 have pentapeptides in common with the spike gp, in a total of 308 occurrences (in all, 462, including multiple occurrences). These numbers certify the existence of an impressive, unexpected level of molecular mimicry between the viral antigen and the cancer-related human proteins. Obvious reasons of space prevent a detailed analysis peptide-by-peptide of the peptide overlap that is given in its entirety in Supplementary Table S2 (available in online version only). Here in text, a snapshot of the peptide sharing is reported and discussed.

### Peptide Sharing between SARS-CoV-2 Spike gp and Tumor-Suppressor Proteins

Table 1 shows data relative to a representative sample of 19 tumor-suppressor proteins and documents that the peptide commonality with the viral antigen amounts to 29 pentapeptides. From a pathological perspective, Table 1 clearly illustrates that even hitting only 19 out of the 294 tumor-suppressor proteins described in Supplementary Table S2 (available in online version only) might equate to induce or enhance carcinogenesis in almost all of the human organs, from brain and liver to lung and bones. Examples of the cancers that might be evoked/potentiated by exposure to SARS-CoV-2 in the next future are T cell acute lymphoblastic leukemia, oligodendrogliomas, breast/ovarian cancers, sarcoma, malignant mesothelioma, B cell chronic lymphocytic leukemia, and non-small cell lung carcinoma, among the others.

**Table 1:** Peptide sharing between SARS-CoV-2 spike gp and a representative sample of 19 tumor-suppressor proteins

Shared peptides	Tumor-suppressor proteins and related cancer diseases a	Refs b
DPFLG	*BC11B. B cell lymphoma/leukemia 11B.* T cell acute lymphoblastic leukemia	27 28
LPPLL, GAGAA, QDVVN, SPDVD	*BICRA. Glioma tumor suppressor candidate.* Oligodendrogliomas	29
EPQII	*BRCA1. Breast cancer type 1 susceptibility protein* . Breast/ovarian cancer	30
SLGAE, LAATK, EPVLK	*BRCA2. Breast cancer type 2 susceptibility protein* . Breast cancer	31
RVVVL	*DCC. Netrin receptor DCC. Deleted in colorectal carcinoma* . Gallbladder cancer	32
YRVVV, SALGK	*DIRA1. GTP-binding protein Di-Ras1. Small GTP-binding tumor suppressor 1* . Lost/downregulated in neural tumors	33
ITDAV	*EXT1. Exostosin-1. Putative tumor suppressor protein EXT1* . Bone tumors	34
ALLAG	*EXT2. Exostosin-2. Putative tumor suppressor protein EXT2* . Bone tumors	34
TLKSF, RLQSL	*IL24. Interleukin-24. Suppression of tumorigenicity 16 protein* . Melanoma	35 36
SKPSK	*LATS1. Large tumor suppressor homolog 1* . Soft tissue sarcoma.	37
ARDLI	*LATS2. Large tumor suppressor homolog 2* . Malignant mesothelioma	38
YSNNS	*MTUS1. Microtubule-associated tumor suppressor 1* . Hepatocellular carcinoma	39
GAGAA	*PLAT2. Phospholipase A and acyltransferase 2* . Gastric cancer	40
GAGAA	*PLAT3. Phospholipase A and acyltransferase 3* . Ovarian carcinoma cells	41
ADAGF, TYVPA	*RBM5. Putative tumor suppressor LUCA15* . Lung cancer	42
RDLPQ, NSVAY	*SCAI. Suppressor of cancer cell invasion* . Downregulated in human tumors	43
LLTDE	*SDS3. Suppressor of defective silencing 3 protein homolog* . Antitumor activity	44
TQSLL, NFKNL, AGAAA	*TASOR. Transcription activation suppressor* . Clear cell renal cell carcinoma	45
LSRLD, GDSSS	*TRI13. B cell chronic lymphocytic leukemia tumor suppressor Leu5* . B cell chronic lymphocytic leukemia. Non-small cell lung carcinoma	46 47

Abbreviations: gp, glycoprotein; SARS-CoV-2, severe acute respiratory syndrome coronavirus 2.

a Tumor-suppressor proteins given by UniProt entry are in italic.

b Further references on cancer diseases are available at UniProt, OMIM, and PubMed.

### Immunologic Potential of the Peptide Sharing between SARS-CoV-2 Spike gp and Tumor-Suppressor Proteins

The gloomy outlook hinted at by the findings described in Table 1 becomes all the more likely in light of the high immunologic potential of the shared peptides. De facto, investigation of IEDB shows that the 29 pentapeptides shared by the spike gp antigen and the 19 tumor-suppressor proteins ( Table 1 ) occur and recur in 150 epitopes derived from SARS-CoV-2 that have been experimentally validated and are cataloged as immunoreactive ( Table 2 ).

**Table 2:** Immunoreactive SARS-CoV-2 spike gp-derived epitopes containing peptides shared between SARS-CoV-2 spike gp and tumor-suppressor human proteins

IEDB ID a	Epitope sequence b	IEDB ID a	Epitope sequence b
36724	litgRLQSL	1329082	ADAGFikqygdclgdia
38831	lQDVVNqnaqalntl	1329083	ADAGFikqygdclgdiaa
51999	qpYRVVVLsf	1329254	demiaqytsALLAG
54725	RLQSLqtyv	1329256	demiaqytsALLAGt
533447	raaeirasanLAATK	1329258	demiaqytsALLAGti
1069290	cTLKSFtvekgiyqt	1329260	demiaqytsALLAGtit
1069445	EPQIIttdntfvsgn	1329323	efqfcnDPFLGvyy
1073938	vqidrlitgRLQSLq	1329325	efqfcnDPFLGvyyh
1074928	ilpdpSKPSK	1329327	efqfcnDPFLGvyyhk
1125063	gltvLPPLL	1329329	efqfcnDPFLGvyyhkn
1309132	nfsqilpdpSKPSKr	1329342	emiaqytsALLAG
1309418	aeirasanLAATKmsecvlg	1329344	emiaqytsALLAGt
1309447	dfggfnfsqilpdpSKPSKr	1329345	emiaqytsALLAGtit
1309450	dplsetkcTLKSFtvekgiy	1329353	EPQIIttdntfvsg
1309451	dsfkeeldkyfknhtSPDVD	1329390	fcnDPFLGvyyh
1309467	fdeddsEPVLKgvklhyt	1329414	fqfcnDPFLGvyy
1309478	gNFKNLrefvfknidgyfki	1329416	fqfcnDPFLGvyyh
1309482	gyqpYRVVVLsfellhapat	1329422	fsqilpdpSKPSKr
1309515	lhrsyltpGDSSSgwtagaa	1329571	idrlitgRLQSLq
1309516	litgRLQSLqtyvtqqlira	1329572	idrlitgRLQSLqt
1309519	lpdpSKPSKrsfiedllfnk	1329595	iqdslsstaSALGKlq
1309523	lssnfgaissvlndiLSRLD	1329597	iraaeirasanLAATK
1309532	ngltvLPPLLTDEmiaqyts	1329606	ITDAVdcaldplse
1309534	nitrfqTLLALhrsyltpgd	1329627	khtpinlvRDLPQg
1309546	pflmdlegkqgNFKNLrefv	1329659	lADAGFikqygdclgdiaa
1309556	qfcnDPFLGvyyhknnkswm	1329710	lpdpSKPSKrsfiedllfnkvt
1309561	qrnfyEPQIIttdntfvsgn	1329762	miaqytsALLAG
1309566	qygdclgdiaARDLIcaqkf	1329764	miaqytsALLAGt
1309567	RDLPQgfsaleplvdlpigi	1329793	ndiLSRLDkveaevq
1309585	sssgwtAGAAAyyvgylqpr	1329940	qidrlitgRLQSLqt
1309589	sygfqptngvgyqpYRVVVL	1329966	qpYRVVVLsfellhapa
1309593	tITDAVdcaldplsetkctl	1329969	qsiiaytmSLGAE
1309599	TYVPAqeknfttapaichdg	1329978	raaeirasanLAATKm
1309605	vsngthwfvtqrnfyEPQII	1330138	staSALGKlQDVVN
1310254	aeNSVAYSNNSiaip	1330167	tdemiaqytsALLAGt
1310284	ARDLIcaqkfngltv	1330169	tdemiaqytsALLAGti
1310303	caqkfngltvLPPLL	1330171	tdemiaqytsALLAGtit
1310362	eldkyfknhtSPDVD	1330209	TLKSFtvekgiyqts
1310392	fgttldskTQSLLiv	1330210	TLKSFtvekgiyqtsn
1310415	fngltvLPPLLTDEm	1330211	TLKSFtvekgiyqtsnf
1310448	gklQDVVNqnaqaln	1330219	tpGDSSSgwtAGAAA
1310586	litgRLQSLqtyvtq	1330220	tpinlvRDLPQg
1310609	lpdpSKPSKrsfied	1330305	vqidrlitgRLQSLqt
1310611	LPPLLTDEmiaqyts	1330306	vqidrlitgRLQSLqtyv
1310747	qpYRVVVLsfellha	1330368	yfkiyskhtpinlvRDLPQ
1310750	qrnfyEPQIIttdnt	1330391	ytsALLAGtit
1310847	titsgwtfGAGAAlq	1330433	diLSRLD
1310947	wtfGAGAAlqipfam	1330434	diLSRLDppeaevq
1311657	ccscgscckfdeddsEPVLKgvkl	1330437	dslsstaSALGKl
1311782	pdpSKPSKrsfiedllfnkvtlad	1330438	dslsstaSALGKlq
1312257	cckfdeddsEPVLKg	1330439	dslsstaSALGKlqdv
1312283	deddsEPVLKgvklh	1330447	EPQIIttdntfvsgnc
1312733	ilpdpSKPSKrsfie	1330456	fsqilpdpSKPSK
1312780	ITDAVdcaldplset	1330457	fsqilpdpSKPSKrs
1313154	miaqytsALLAGtit	1330463	gfnfsqilpdpSKPSKr
1313286	pinlvRDLPQgfwal	1330487	ilpdpSKPSKr
1313756	TLKSFtvek	1330489	iqdslsstaSALGKl
1313930	vTYVPAqeknfttap	1330490	iqdslsstaSALGKlqd
1314170	ADAGFikqy	1330515	lADAGFikqy
1315180	aYSNNSiai	1330551	pSKPSKrsf
1316068	etkcTLKSF	1330552	pSKPSKrsfi
1316945	fsqilpdpSKPSKrsfie	1330557	qilpdpSKPSKr
1318209	hvTYVPAqek	1330589	slsstaSALGKlq
1320443	lgaeNSVAY	1330597	sqilpdpSKPSK
1321084	LPPLLTDEm	1330598	sqilpdpSKPSKr
1323467	qpYRVVVL	1330623	tpinlvRDLPQgfs
1323750	rasanLAATK	1330624	tpinlvRDLPQgfsa
1323919	RLQSLqty	1330625	tpinlvRDLPQgfsalepl
1324353	setkcTLKSF	1331139	cnDPFLGvy
1325536	tlADAGFik	1332424	itgRLQSLqty
1327824	wtAGAAAyy	1332664	LLTDEmiaqy
1327836	wtfGAGAAl	1334122	TYVPAqeknft
1328800	ytmslgaeNSVAY	1334394	yqpYRVVVL
1328800	ytmSLGAEnsvay	1334452	alhrsyltpGDSSSg
1329076	aaeirasanLAATK	1334473	NSVAYSNNSiaiptnft

Abbreviations: gp, glycoprotein; IEDB, Immune Epitope Database; SARS-CoV-2, severe acute respiratory syndrome coronavirus 2.

a Epitopes listed according to the IEDB ID number.

b Shared sequences given are capitalized.

In essence, Table 2 factually supports the possibility that cross-reactions can be triggered by SARS-CoV-2 infection/active immunization and hit human proteins related to carcinogenesis. Very much this conclusion applies when considering that the extent of the potential immunologic cross-reactivity as well as the spectrum of potentially inducible tumors may be exponentially higher in light of the fact that Tables 1 and 2 refer to the peptide commonality involving only a tiny part (19 out of 294) of the human proteins that—if altered—may lead to cancer (see Supplementary Table S2 [available in online version only] for the peptide sharing in its totality).

### Potential Immunologic Imprint

The 29 pentapeptides common to SARS-CoV-2 spike gp and tumor-suppressor proteins ( Table 1 ) are not only present in immunoreactive epitopes ( Table 2 ) but, in addition, almost all of them (24 out of 29) are also present in microbial organisms such as *Bordetella pertussis* , *C. diphtheriae* , *C. tetani* , *H. influenzae* , and *N. meningitides* ( Table 3 ). That is, most of the shared peptides are also present in pathogens that an individual possibly encountered during his life because of infections and/or vaccinal routes.

**Table 3:** Occurrences in microbial organisms of pentapeptides common to SARS-CoV-2 spike gp, human proteins related to cancer, and SARS-CoV-2 spike gp-derived epitopes

Organism	Shared peptides
*Bordetella pertussis*	ADAGF, AGAAA, ALLAG, GAGAA, ITDAV, RLQSL, SLGAE, SPDVD, TYVPA
*Clostridium tetani*	AGAAA, LAATK, LLTDE, YSNNS
*Corynebacterium diphtheriae*	AGAAA, ALLAG, EPQII, GAGAA, ITDAV, SALGK, YRVVV
*Haemophilus influenzae*	AGAAA, GAGAA, LLTDE, LPPLL, LSRLD, NFKNL, NSVAY, RDLPQ, RLQSL, RVVVL, SALGK, SLGAE, TLKSF, TQSLL, YSNNS
*Neisseria meningitides*	AGAAA, ALLAG, EPVLK, GAGAA, LLTDE, LPPLL

Abbreviations: gp, glycoprotein; SARS-CoV-2, severe acute respiratory syndrome coronavirus 2.

Such interpathogen peptide commonality introduces the immunologic memory as a factor capable of enhancing the extent of the immune cross-reactive response against the tumor-suppressor proteins. That is, as already described since 1947, 48 49 the immune system does not induce ex novo primary responses toward a recent infection. Rather, the immune system recalls, amplifies, and intensifies preexisting memory responses toward past infections. In this way, what should have been a primary response to a recent infection is transformed into an anamnestic, secondary, and magnified response to past infections. Simply put, as already discussed in previous reports, 50 51 52 53 54 55 the early history of the individual's infections/vaccinations dictates the immune outcomes of any successive infections/vaccinations.

The immunologic imprint phenomenon has its molecular foundations in the massive peptide sharing that characterizes microbial and human proteins 17 56 57 and of which Table 3 is an example. The implications are noteworthy. In the case object, following exposure to SARS-CoV-2 by infection or vaccination, the expected primary response to the virus can turn into a secondary response to previously encountered pathogens against which the immune system already reacted and of which has stored an immunologic memory, that is, the microbial organisms reported in Table 3 . However, the previously encountered pathogens are no more present in the human organism, so that the anamnestic immune response triggered by the exposure to SARS-CoV-2 by infection or vaccination ends to divert onto available immune determinants that, in the present case, are the common determinants present in the tumor-suppressor human proteins. Pathologically, one has to consider that usually an anamnestic secondary immune response is characterized by high avidity and high affinity, besides being quantitatively relevant. Therefore, as a final result, exposure to SARS-CoV-2 by infection and/or vaccination can trigger immediate and violent cross-reactive attacks against the proteins that protect the human being from carcinogenesis.

## Conclusion

The findings described in Tables 1 to 3 and Supplementary Table S2 (available in online version only) indicate that molecular mimicry and cross-reactivity between peptides common to SARS-CoV-2 and tumor-related proteins might cause/contribute to cancer epidemics worldwide in the next future. The potential cancer risk might be enhanced by immunologic imprinting phenomena, given the fact that the comparative analyses shown in Table 3 indicate the possibility that a preexisting immune response to previously encountered pathogens could be magnified and intensified following SARS-CoV-2 infection/active immunization. These data are disturbing and invite to immediately intensify clinical surveillance in oncology and to undertake rigid cancer prevention actions, including healthy lifestyle and continuous controls. It will be vital to formulate/implement actions that contemplate fast and safe procedures for clinical trials, development of specific and reliable tumor markers for diagnosis, accurate follow-up of treatments, and, administratively, medical health records, detailed registries, biobanks, health surveys, and coordinated observational studies. Never before do all the recommendations of the European plan for the fight against cancer appear current and necessary. 58 De facto, tumors appear to be the predominant pathologies that will populate the post pandemic long COVID-19.
