# Supplementary material for: From Anti-Severe Acute Respiratory Syndrome Coronavirus 2 Immune Response to Cancer Onset via Molecular Mimicry and Cross-Reactivity
Source: Glob Med Genet. 2021 Sep 7;8(4):176–82. doi: 10.1055/s-0041-1735590 (PMC8635832; doi:10.1055/s-0041-1735590)
Supplement: Supplementary file 1 — Supplementary Material [file 10-1055-s-0041-1735590-s2100033.pdf]

Supplementary Table S1 List of 782 human proteins (in)directly linked to cancer and obtained from UniProtKB database (www.uniprot.org) using "tumor suppressor" as keywords

2A5G; 2AAA; 2AAB; ACER2; ACK1; ACL6A; ACTB; AGAP2; AGRA2; AGRA3; AGRB1; AHRR; AHSA1; AIM2; AKT1; AMER1; AMER2; AMRP; AN32A; ANM3; ANM5; ANM6; AP2A; AP2C; APAF; APC; APCL; ARF; ARHG4; ARI3B; ARL11; ASC; ASPP2; ATF2; ATG7; ATM; ATR; AXIN1; B2L14; B4GA1; BANP; BAP1; BARD1; BAX; BC11B; BCCIP; BCL10; BCL7B; BECN1; BHE40; BICRA; BICRL; BIN1; BIRC2; BIRC3; BLCAP; BMI1; BMR1A; BNI3L; BORC5; BRCA1; BRCA2; BRD1; BRD4; BRD7; BRD8; BRD9; BRE1A; BRMS1; BRNP1; BRPF1; BRPF3; BTG2; BTK; BUB1B; C56D2; CA052; CA2D3; CADM1; CADM3; CADM4; CARF; CAVN3; CBL; CBPC2; CBX2; CBX4; CBX7; CBX8; CCAR2; CCL24; CCND1; CCND3; CCNE1; CDC23; CDC37; CDC73; CDHR2; CDK10; CDK4; CDK6; CDKA1; CDN1B; CDN1C; CDN2A; CDN2B; CDN2C; CDN2D; CERS2; CHD5; CHDH; CHFR; CHK1; CHK2; CI014; CIA2A; CIAO1; CIP2A; CJ090; CK5P3; CKAP4; CLCA1; CLCA2; CMTA1; CMTA2; CNDP2; CO4A1; COMD1; CRBL2; CRCM; CREB3; CRTAM; CSC2A; CSK21; CSK23; CSMD1; CSN5; CSRN1; CTCF; CTCFL; CTDSL; CTIP; CTNB1; CUL2; CYLD; DAB2; DAB2P; DAPK1; DAPK3; DAXX; DBF4A; DCC; DDB1; DDIT4; DDT4L; DDX17; DDX3X; DDX5; DDX58; DEC1; DEMA; DEPD5; DERPC; DFFB; DI3L2; DIDO1; DIRA1; DIRA3; DLEC1; DLG1; DLG3; DLG4; DLP1; DMAP1; DMBT1; DMRT1; DMTF1; DNJA3; DNJB6; DNMT1; DOCK8; DPH1; DRC6; DUS16; DVL3; E2F1; E2F2; E41L3; E4F1; EAF2; EAF6; EFNA1; EGFR; EGLN1; EHF; EHMT1; EHMT2; EI24; EIF3E; ELOB; ELOC; EM55; ENOA; EP300; EPB41; EPC1; EPHB2; ERRFI; ESR1; ESR2; EXT1; EXT2; EXTL2; EXTL3; EZH2; F107A; F10A1; F10A5; F120A; F210B; FA83D; FABP7; FANCG; FANCM; FAT1; FAT3; FAT4; FBLN1; FBW1A; FBX31; FBXL2; FBXW5; FBXW7; FES; FETUB; FGL1; FHIT; FL2D; FLCN; FOCAD; FOXC1; FOXD1; FOXO1; FOXP1; FRK; FRMD3; FUMH; FZD5; GBRAP; GBRL2; GCR; GGNB2; GKAP1; GKN2; GNTK; GP15L; GPS2; GRAA; GRB2; GRK5; GSDME; HABP2; HACE1; HAVR2; HDAC1; HDAC7; HEMK1; HERC5; HGS; HIC1; HIC2; HIF1A; HIF3A; HINT1; HIPK2; HM20B; HOP; HRG; HS90A; HTAI2; HTRA3; HUWE1; HYAL1; HYAL2; HYAL3; I23O1; IBPL1; IF16; IF4A1; IF4E; IFIX; IFRD2; IGF1R; IKKE; IL24; IL37; ING1; ING2; ING3; ING4; ING5; INP4B; INSM2; INT6; IRF1; IRF3; ITIH5; JADE1; JADE2; JADE3; JHD2C; KANK1; KAT5; KAT6A; KAT6B; KAT7; KCD11; KCD21; KCNRG; KCTD6; KDM3B; KDM5B; KI13B; KIBRA; KIF1B; KIF3A; KIF3B; KISS1; KISSR; KIT; KLF4; KLF6; KLK10; KMT5A; KPCD; KPCG; KPCI; KPYM; KS6A1; KS6A2; KS6A3; L2GL1; L2GL2; LACTB; LARG1; LARG2; LATS1; LATS2; LEO1; LEU1; LEU7; LGI1; LGR6; LIMD1; LIN9; LMBL1; LN28A; LN28B; LPP; LPPRC; LRP12; LRP1B; LYPD1; LZTL1; LZTS1; LZTS2; LZTS3; M2OM; M3K20; M3K7; MAD1; MAF; MAFB; MAGI2; MAGI3; MANS1; MAOM; MAP1S; MAPK3; MAPK5; MAST2; MAVS; MCA3; MCM2; MCTS1; MDM2; MED28; MEN1; MENT; MEOX2; MERL; MERTK; MFHA1; MINY3; MIO; MIRO1; MITF; MK01; MK03; MK12; MK14; MK15; MLH1; MLP3A; MMP14; MMP2; MMP9; MN1; MO4L1; MO4L2; MOAP1; MOD5; MP2K4; MPP2; MPP3; MRGBP; MSH2; MSTRO; MTA1; MTAP; MTG16; MTG8R; MTOR; MTSS1; MTUS1; MTUS2; MUC1; MUTYH; MXI1; MY18B; MZF1; NAA80; NACC1; NAPEP; NBL1; NDKA; NDKB; NDRG1; NDRG2; NEB2; NEDD8; NEMF; NEMO; NEUL1; NF1; NFKB1; NFKB2; NISCH; NIT1; NIT2; NKX31; NMDE2; NOL7; NOP53; NPAS2; NPM; NPRL2; NPRL3; NSA2; NSE3; NSG1; NSMA2; OGR1; OPCM; OVCA2; P53; P5I13; P63; P73; PA2G4; PACER; PAF1; PAF15; PAI1; PALB2; PANO1; PARK7; PAWR; PB1; PCGF2; PCGF6; PCM1; PDCD4; PEG3; PERP; PEX19; PFD3; PFD5; PGBD5; PGDH; PGFRL; PHC1; PHC2; PHC3; PHF24; PHLA3; PHLP1; PHLP2; PIAS1; PIAS2; PIN1; PININ; PINX1; PKHG2; PKHO1; PLAL1; PLAT1; PLAT2; PLAT3; PLAT4; PLK1; PLK2; PLPP5; PML; PMS1; PMS2; PNM8A; PPAP; PPP5; PRDM2; PRDM4; PRDM5; PRDX1; PRKN; PRR5; PTC1; PTC2; PTEN; PTN14; PTPRJ; PTPRK; PUF60; R51A1; RAD54; RAF1; RAI3; RAP1A; RASA1; RASEF; RASF1; RASF2; RASF3; RASF4; RASF5; RASFA; RASH; RASK; RB; RBBP4; RBBP7; RBCC1; RBL1; RBL2; RBM5; RBM6; RBM8A; RBMX; RBP2; RBX1; RECK; RFIP4; RGPA1; RGS16; RHG07; RHG20; RHG29; RHG35; RHOB; RHXF2; RING1; RING2; RL10; RMP; RN126; RN135; RN167; RNF43; RNZ1; RPH3L; RRAGA; RRAGB; RRAGC; RRAGD; RS29; RSLAA; RSPO1; RSPO2; RSPO3; RUNX3; RUVB1; RUVB2; S10A2; S10A6; S22AI; S26A3; S38A3; SAPC2; SASH1; SATB1; SAV1; SC5A8; SCAI; SCRIB; SDHA; SDS3; SEC13; SEH1; SEM3B; SEPR; SESN1; SET; SETB1; SETD2; SGSM3; SHAN2; SHC1; SIAH1; SIK1; SIR1; SIR2; SIR4; SMAD2; SMAD4; SMAD5; SMAD6; SMCA4; SMO; SMRC1; SMRD1; SMRD3; SMYD2; SN12L; SNF5; SNW1; SP1; SP100; SPB5; SQSTM; SSXT; ST134; ST14; ST18; ST20; ST65G; ST7; ST7L; STA13; STA5B; STAT3; STEA3; STIM1; STIP1; STK11; STK3; STK4; STRAA; SUFU; SUMO1; SUMO2; SUSD2; SUSD6; SYNP2; T106A; T132E; T184C; T53I1; TAB2; TAB3; TACC2; TAF1; TAF1C; TARG1; TASOR; TBK1; TBRG1; TCAL7; TCF7; TCHP; TCP1L; TEBP; TEFF1; TES; TET2; TF2H1; TF65; TF7L2; TFE3; TFF1; TGO1; THEM4; TIF1A; TIF1B; TIG1; TIRAP; TM115; TM127; TM158; TMM8B; TMUB1; TNK1; TOPB1; TOPK; TOPRS; TP53B; TPA; TRADD; TRAF2; TRAF6; TRAIP; TRI13; TRI35; TRI36; TRI37; TRI59; TRIP6; TRIPC; TRM9B; TSC1; TSC2; TTP; TUSC1; TUSC2; TUSC3; TUT7; TXNIP; TZAP; U2QL1; UB2V1; UBAP1; UBC9; UBE2O; UBE3A; UBP11; UBP12; UBP20; UBP28; UBP33; UBP7; UBR5; UBXN1; UHRF1; UHRF2; UN93A; UROK; USBP1; USH1C; UVRAG; VHL; VILL; VISTA; VMA5A; VRK1; WDR24; WDR48; WDR59; WNT4; WNT5A; WT1; WWOX; XAF1; XPO1; XRN1; YAP1; YETS4; ZBT7A; ZBT7C; ZC12D; ZDH14; ZDH17; ZDHC2; ZFHX3; ZGPAT; ZMY10; ZMY11; ZN217; ZN276; ZN304; ZN652; ZNRF3; ZO1; ZSC32; ZZEF1

Note: Proteins listed by UniProt entry.

 © 2021. The Author(s).

Supplementary Table S2 Pentapeptide sharing between SARS-CoV-2 spike gp and human proteins (in)directly related to cancer

VLLPL	2A5G. Serine/threonine-protein phosphatase 2A 56 kDa regulatory subunit gamma isoform
SQILP	2AAA. Serine/threonine-protein phosphatase 2A 65 kDa regulatory subunit A α isoform
KLIAN	ACL6A. Actin-like protein 6A
AGAAA, ASALG	AGRA2. Adhesion G protein-coupled receptor A2
QAGST	AGRA3. Adhesion G protein-coupled receptor A3
HAPAT	AHSA1. Activator of 90 kDa heat shock protein ATPase homolog 1
SPRRA	AMER1. APC membrane recruitment protein 1
SVLHS, EELDK	AMRP. Alpha-2-macroglobulin receptor-associated protein
DGVYF	ANM3. Protein arginine N-methyltransferase 3
VLYQD	ANM5. Protein arginine N-methyltransferase 5
SSVLH	ANM6. Protein arginine N-methyltransferase 6
NITNL	APAF. Apoptotic protease-activating factor 1
DSLSS	APC. Adenomatous polyposis coli protein
FTVEK	APCL. Adenomatous polyposis coli protein 2
GTNTS	ARI3B. AT-rich interactive domain-containing protein 3B
SPTKL, TEVPV	ASPP2. Apoptosis-stimulating of p53 protein 2
KGCCS	ATM. Serine-protein kinase ATM
PGDSS, ALNTL	ATR. Serine/threonine-protein kinase ATR
NSPRR	B4GA1. Beta-1,4-glucuronyltransferase 1
ITGRL, QQLIR	BAP1. Ubiquitin carboxyl-terminal hydrolase BAP1
SFELL	BARD1. BRCA1-associated RING domain protein 1
DPFLG	BC11B. B cell lymphoma/leukemia 11B
VFNAT, EDDSE	BECN1. Beclin-1
LPPLL, GAGAA, QDVVN, SPDVD	BICRA. BRD4-interacting chromatin-remodeling complex-associated protein
ISVTT	BIN1. Myc box-dependent-interacting protein 1
TEIYQ, LGFIA	BMR1A. Bone morphogenetic protein receptor type-1A
EPQII	BRCA1. Breast cancer type 1 susceptibility protein
SLGAE, LAATK, EPVLK	BRCA2. Breast cancer type 2 susceptibility protein
KLQDV	BRD4. Bromodomain-containing protein 4
KVGGN, **TGRLQS**	BRD7. Bromodomain-containing protein 7
KEELD	BRD8. Bromodomain-containing protein 8
GDSSS	BRD9. Bromodomain-containing protein 9
**SLLIVN**, SVLYN	BRE1A. E3 ubiquitin-protein ligase BRE1A
**AVDCAL**	BRPF1. Peregrin
**QTLLAL**	BUB1B. Mitotic checkpoint serine/threonine-protein kinase BUB1 β
SPRRA, SALLA	CA2D3. Voltage-dependent calcium channel subunit α-2/delta-3
AVEQD	CARF. CDKN2A-interacting protein
LREFV, ASALG	CBL. E3 ubiquitin-protein ligase CBL
PGDSS, TFLLK, LPDPS	CBX8. Chromobox protein homolog 8
RSVAS	CCAR2. Cell cycle and apoptosis regulator protein 2
KEELD	CDC37. Hsp90 co-chaperone Cdc37
PYRVV	CDC73. Parafibromin
AYSNN	CDHR2. Cadherin-related family member 2
GQSKR	CDK10. Cyclin-dependent kinase 10

(*Continued*)

Supplementary Table S2 (*Continued*)

LVRDL, LPPLL, ILSRL	CHD5. Chromodomain-helicase-DNA-binding protein 5
NNSYE	CHFR. E3 ubiquitin-protein ligase CHFR
DEVRQ	CI014. Putative uncharacterized protein encoded by LINC00032
QDVNC	CIAO1. Probable cytosolic iron-sulfur protein assembly protein CIAO1
KIADY, LNESL	CIP2A. Protein CIP2A
FNKVT	CLCA1. Calcium-activated chloride channel regulator 1
SALEP, RVVVL	CMTA2. Calmodulin-binding transcription activator 2
ELGKY	COMD1. COMM domain-containing protein 1
SLSST, SSTAS	CRCM. Colorectal mutant cancer protein
EHVNN	CSK21. Casein kinase II subunit α
EHVNN	CSK23. Casein kinase II subunit α 3
**TLDSKV**, SIVRF	CSMD1. CUB and sushi domain-containing protein 1
SFELL, SSTAS	CSRN1. Cysteine/serine-rich nuclear protein 1
VLPPL	CTDSL. CTD small phosphatase-like protein
ALDPL	DAB2. Disabled homolog 2
IQDSL	DAPK1. Death-associated protein kinase 1
RVVVL	DCC. Netrin receptor DCC
**SFELLH**, LDITP, LGAEN	DDB1. DNA damage-binding protein 1
SSANN, GAAAY	DDX17. Probable ATP-dependent RNA helicase DDX17
GGNYN	DDX3X. ATP-dependent RNA helicase DDX3X
FFSNV, VEKGI	DDX58. Antiviral innate immune response receptor RIG-I
VITPG, NGIGV	DEPD5. GATOR complex protein DEPDC5
**NSASFS**	DERPC. Decreased expression in renal and prostate cancer protein
TQNVL	DI3L2. DIS3-like exonuclease 2
SSTAS	DIDO1. Death-inducer obliterator 1
YRVVV	DIRA1. GTP-binding protein Di-Ras1
YRVVV	DIRA3. GTP-binding protein Di-Ras3
LDSKV, DCLGD	DLEC1. Deleted in lung and esophageal cancer protein 1
QVKQI	DLG1. Disks large homolog 1
PGQTG	DMRT1. Doublesex- and mab-3-related transcription factor 1
**AALQIP**	DMTF1. Cyclin-D-binding Myb-like transcription factor 1
PREGV	DNJA3. DnaJ homolog subfamily A member 3, mitochondrial
LEPLV, TQLNR	DOCK8. Dedicator of cytokinesis protein 8
VTLAD	DVL3. Segment polarity protein disheveled homolog DVL-3
SASFS	E41L3. Band 4.1-like protein 3
GAGAA	E4F1. Transcription factor E4F1
**NIIRGW**	EAF6. Chromatin modification-related protein MEAF6
**TAGAAL**	EGFR. Epidermal growth factor receptor
GDEVR	EGLN1. Egl nine homolog 1
LDSKT	EHF. ETS homologous factor
**DEDDSE**	EHMT1. Histone-lysine N-methyltransferase EHMT1
AGAAA	EHMT2. Histone-lysine N-methyltransferase EHMT2
NLLLQ	EI24. Etoposide-induced protein 2.4 homolog
LQSLQ	EIF3E. Eukaryotic translation initiation factor 3 subunit E

 © 2021. The Author(s).

Supplementary Table S2 (*Continued*)

APATV	ELOB. Elongin-B
LPDPS	EP300. Histone acetyltransferase p300
YTSAL	EPHB2. Ephrin type-B receptor 2
RISNC, LPPLL	ERRFI. ERBB receptor feedback inhibitor 1
SALGK	ESS2. Splicing factor ESS-2 homolog
ITDAV	EXT1. Exostosin-1
ALLAG	EXT2. Exostosin-2
GQTGK	EZH2. Histone-lysine N-methyltransferase EZH2
SSTAS, CDVVI	F120A. Constitutive coactivator of PPAR-gamma-like protein 1
LFLPF, IGAGI	FANCM. Fanconi anemia group M protein
VFRSS, SGTNG, **SLSSTA**	FAT1. Protocadherin Fat 1
DSSSG, **SLSSTA**, MVTIM	FAT3. Protocadherin Fat 3
GTNGT, LVRDL, DSSSG, RTFLL, LTGTG, TSALL	FAT4. Protocadherin Fat 4
FGEVF, TLEIL	FES. Tyrosine-protein kinase Fes/Fps
GPKKS	FLCN. Folliculin
LSSTA, RLNEV	FOCAD. Focadhesin
GAGAA	FOXC1. Forkhead box protein C1
RISNC	FOXD1. Forkhead box protein D1
QLTPT, VASQS	GCR. Glucocorticoid receptor
ALEPL, KNKCV	GGNB2. Gametogenetin-binding protein 2
HVSGT, QQLIR	GSDME. Gasdermin-E
KLIAN	HABP2. Hyaluronan-binding protein 2
**IGAGICA**, VTLAD	HAVR2. Hepatitis A virus cellular receptor 2
VLLPL, TLLAL, DPLSE	HDAC7. Histone deacetylase 7
TFGAG	HERC5. E3 ISG15–protein ligase HERC5
GYQPY	HGS. Hepatocyte growth factor-regulated tyrosine kinase substrate
MDLEG	HIC2. Hypermethylated in cancer 2 protein
LDSKT	HIF1A. Hypoxia-inducible factor 1-α
GCVIA, QPYRV, VSVIT	HIPK2. Homeodomain-interacting protein kinase 2
DSSSG	HM20B. SWI/SNF-related matrix-associated actin-dependent regulator of chromatin subfamily E member 1-related
VLTES	HTRA3. Serine protease HTRA3
LLPLV, LADAG, DEDDS **STASAL**, EAEVQ,	HUWE1. E3 ubiquitin-protein ligase HUWE1
QLSSN	IF4E. Eukaryotic translation initiation factor 4E
TLADA, ASALG	IFRD2. Interferon-related developmental regulator 2
TLKSF, RLQSL	IL24. Interleukin-24
SALLA, LNTLV	INP4B. Inositol polyphosphate 4-phosphatase type II
VLLPL	INT6. Integrator complex subunit 6
KTSVD, LGKYE	ITIH5. Inter-α-trypsin inhibitor heavy chain H5
RSVAS	JHD2C. Probable JmjC domain-containing histone demethylation protein 2C
QTLLA, KIQDS	KANK1. KN motif and ankyrin repeat domain-containing protein 1
SVTTE, LSSTA, DEDDS	KAT6B. Histone acetyltransferase KAT6B
EVFNA	KCD21. BTB/POZ domain-containing protein KCTD21

(*Continued*)

 © 2021. The Author(s).

Supplementary Table S2 (*Continued*)

QTLLA	KDM5B. Lysine-specific demethylase 5B
GDSSS, EELDK	KI13B. Kinesin-like protein KIF13B
LLAGT, LAGTI	KIF1B. Kinesin-like protein KIF1B
ADYNY	KIF3A. Kinesin-like protein KIF3A
LALHR, **ANLAAT**	KISSR. KiSS-1 receptor
SFSTF, GAGAA	KLF4. Krueppel-like factor 4
VKLHY	KS6A1. Ribosomal protein S6 kinase α-1
VKLHY	KS6A2. Ribosomal protein S6 kinase α-2
VKLHY	KS6A3. Ribosomal protein S6 kinase α-3
DISGI	L2GL1. Lethal(2) giant larvae protein homolog 1
HADQL	LARG2. LARGE xylosyl- and glucuronyltransferase 2
SKPSK	LATS1. Serine/threonine-protein kinase LATS1
ARDLI	LATS2. Serine/threonine-protein kinase LATS2
LQELG	LGR6. Leucine-rich repeat-containing G-protein coupled receptor 6
FIEDL	LIMD1. LIM domain-containing protein 1
GAGIC	LN28A. Protein lin-28 homolog A
GKLQD	LPPRC. Leucine-rich PPR motif-containing protein, mitochondrial
NGLTV	LRP1B. Low-density lipoprotein receptor-related protein 1B
TNTSN, STASA	M3K7. Mitogen-activated protein kinase kinase kinase 7
SLSST	MAF. Transcription factor Maf
ALHRS	MAGI2. Membrane-associated guanylate kinase, WW and PDZ domain-containing protein 2
LPDPS	MAP1S. Microtubule-associated protein 1S
GDSTE	MAST2. Microtubule-associated serine/threonine-protein kinase 2
EGKQG	MAVS. Mitochondrial antiviral-signaling protein
SNIIR, TQLNR, VLYEN	MIO. GATOR complex protein MIOS
VNNTV	MIRO1. Mitochondrial Rho GTPase 1
SNLLL	MK01. Mitogen-activated protein kinase 1
LVKNK, VLPPL	MPP3. MAGUK p55 subfamily member 3
NQVAV, AARDL	MSH2. DNA mismatch repair protein Msh2
ITPGT	MTAP2. Microtubule-associated protein 2
SSTAS	MTG16. Protein CBFA2T3
VYDPL	MTOR. Serine/threonine-protein kinase mTOR
YSNNS	MTUS1. Microtubule-associated tumor suppressor 1
TVEKG, LPDPS, GFIKQ, SSVLN	MY18B. Unconventional myosin-XVIIIb
**AGAGAA**	MZT1. Mitotic-spindle organizing protein 1
LPLVS	NACC1. Nucleus accumbens-associated protein 1
IPIGA, **KEELDK**	NAPEP. N-acyl-phosphatidylethanolamine-hydrolyzing phospholipase D
LDITP	NDRG1. Protein NDRG1
YLTPG, LTPGD	NEMF. Nuclear export mediator factor NEMF
LALHR, LVKQL, VLGQS, PLQPE, SCLKG	NF1. Neurofibromin
LVRDL	NFKB1. Nuclear factor NF-kappa-B p105 subunit
ALLAG	NFKB2. Nuclear factor NF-kappa-B p100 subunit
SLSST	NISCH. Nischarin
SFSTF	NIT2. Omega-amidase NIT2

 © 2021. The Author(s).

Supplementary Table S2 (*Continued*)

KQLSS	NKX31. Homeobox protein Nkx-3.1
ANLAA	NMDE2. Glutamate receptor ionotropic, NMDA 2B
KVTLA	NPM. Nucleophosmin
NCVAD	OGR1. Ovarian cancer G-protein coupled receptor 1
FSALE, AQALN	OVCA2. Esterase OVCA2
NVLYE	PA2G4. Proliferation-associated protein 2G4
SLSST	PACER. Protein associated with UVRAG as autophagy enhancer
LQYGS	PB1. Protein polybromo-1
GTNTS	PCGF2. Polycomb group RING finger protein 2
PLSET	PCGF6. Polycomb group RING finger protein 6
YENQK	PEG3. Paternally expressed gene 3 protein
**QTLEIL**	PFD3. Prefoldin subunit 3
PLVSS, ESNKK	PHC3. Polyhomeotic-like protein 3
TEVPV, NFSQI	PHLP2. PH domain leucine-rich repeat-containing protein phosphatase 2
**LVKQLS**, KQLSS	PIAS1. E3 SUMO-protein ligase PIAS1
TEVPV	PININ. Pinin
SLSST, RASAN, **LQELGK**	PKHG2. Pleckstrin homology domain-containing family G member 2
FCGKG	PKHO1. Pleckstrin homology domain-containing family O member 1
GAGAA	PLAT2. Phospholipase A and acyltransferase 2
GAGAA	PLAT3. Phospholipase A and acyltransferase 3
LLALH	PML. Protein PML
LFNKV	PMS1. PMS1 protein homolog 1
SPRRA, KNLNE	PNM8A. Paraneoplastic antigen-like protein 8°
VYDPL	PPAP. Prostatic acid phosphatase
LPPLL, GIGVT, AISSV	PRDM2. PR domain zinc finger protein 2
IITTD, EDDSE	PRDM4. PR domain zinc finger protein 4
PQGFS	PRR5. Proline-rich protein 5
LVLLP	PTC1. Protein patched homolog 1
LVLLP	PTC2. Protein patched homolog 2
IDRLI	PTPRJ. Receptor-type tyrosine-protein phosphatase eta
SKRSF, IVMVT	PTPRK. Receptor-type tyrosine-protein phosphatase kappa
LEILD	RAP1A. Ras-related protein Rap-1A
AGAAA	RASA1. Ras GTPase-activating protein 1
QSKRV	RASEF. Ras and EF-hand domain-containing protein
NLAAT	RASF5. Ras association domain-containing protein 5
SSTAS	RASFA. Ras association domain-containing protein 10
RKSNL, ISTEI	RB. Retinoblastoma-associated protein
LVSSQ, GDSSS, LFRKS, SFCTQ	RBCC1. RB1-inducible coiled-coil protein 1
LQDVV	RBL1. Retinoblastoma-like protein 1
ADAGF, TYVPA	RBM5. RNA-binding protein 5
GFNFS, KLQDV	RBP2. E3 SUMO-protein ligase RanBP2
AVEQD	RGPA1. Ral GTPase-activating protein subunit α-1
LDSKV, LFRKS, TEIYQ, SLSST, NLAAT	RHG07. Rho GTPase-activating protein 7
AKNLN	RHG29. Rho GTPase-activating protein 29

(*Continued*)

 © 2021. The Author(s).

Supplementary Table S2 (*Continued*)

SSTAS	RHG35. Rho GTPase-activating protein 35
DSSSG, SPRRA	RNF43. E3 ubiquitin-protein ligase RNF43
LADAG	RRAGC. Ras-related GTP-binding protein C
LADAG	RRAGD. Ras-related GTP-binding protein D
LNRAL	RUVB2. RuvB-like 2
IAGLI	S26A3. Chloride anion exchanger
CSFGG	SASH1. SAM and SH3 domain-containing protein 1
QDKNT	SATB1. DNA-binding protein SATB1
RDLPQ, NSVAY	SCAI. Protein SCAI
RLQSL	SCRIB. Protein scribble homolog
AGCLI	SDHA. Succinate dehydrogenase (ubiquinone) flavoprotein subunit, mitochondrial
LLTDE	SDS3. Sin3 histone deacetylase corepressor complex component SDS3
NKVTL	SEC13. Protein SEC13 homolog
AGAAA	SEM3B. Semaphorin-3B
EIDRL, IDRLN, DRLNE	SET. Protein SET
LTGTG, ILDIT, IYKTP, STASA	SETB1. Histone-lysine N-methyltransferase SETDB1
FASVY, IEDLL	SGSM3. Small G protein signaling modulator 3
LQPRT, DSFVI	SHAN2. SH3 and multiple ankyrin repeat domains protein 2
DPFLG	SIR2. NAD-dependent protein deacetylase sirtuin-2
PATVC	SMAD2. Mothers against decapentaplegic homolog 2
DLPQG, AGAAL	SMAD6. Mothers against decapentaplegic homolog 6
DSSSG, EDDSE	SMCA4. Transcription activator BRG1
AGAAA	SMRD1. SWI/SNF-related matrix-associated actin-dependent regulator of chromatin subfamily D member 1
GAGAA	SMRD1. SWI/SNF-related matrix-associated actin-dependent regulator of chromatin subfamily D member 1
FLGVY, TEILP, DSEPV	SN12L. Schlafen family member 12-like
LVSSQ	SP1. Transcription factor Sp1
DPSKP, LQSLQ	SQSTM. Sequestosome-1
PLVSS, LFRKS, TEIYQ	STA13. StAR-related lipid transfer protein 13
TLLAL	STEA3. Metalloreductase STEAP3
PPLLT	STIM1. Stromal interaction molecule 1
GLIAI	STIP1. Stress-induced-phosphoprotein 1
NCVAD	STK3. Serine/threonine-protein kinase 3
NCVAD	STK4. Serine/threonine-protein kinase 4
SLGAE	SYNP2. Synaptopodin-2
SALLA	T132E. Transmembrane protein 132E
YSTGS, GDIAA	TACC2. Transforming acidic coiled-coil-containing protein 2
**YLTPGD**, SALLA	TAF1C. TATA box-binding protein-associated factor RNA polymerase I subunit C
TQSLL, NFKNL, AGAAA	TASOR. Protein TASOR
SPRRA	TBKB1. TANK-binding kinase 1-binding protein 1
EPLVD	TBRG1. Transforming growth factor β regulator 1
STNLV	TF2H1. General transcription factor IIH subunit 1
DKVFR, LYNSA	TGO1. Transport and Golgi organization protein 1 homolog
LDKVE	THEM4. Acyl-coenzyme A thioesterase THEM4

 © 2021. The Author(s).

Supplementary Table S2 (*Continued*)

PPAYT	TM127. Transmembrane protein 127
**VTLADAG**	TNK1. Nonreceptor tyrosine-protein kinase TNK1
**LHRSYL**	TOPB1. DNA topoisomerase 2-binding protein 1
NVVIK	TOPK. Lymphokine-activated killer T cell-originated protein kinase
SLGAE, QDSLS, KQLSS,**DLGDIS**	TP53B. TP53-binding protein 1
LSRLD	TRI13. E3 ubiquitin-protein ligase TRIM13
**SFKEELD**	TRI35. Tripartite motif-containing protein 35
DVDLG	TRI36. E3 ubiquitin-protein ligase TRIM36
VTQQL	TRI37. E3 ubiquitin-protein ligase TRIM37
GAALQ	TRIP6. Thyroid receptor-interacting protein 6
LVDLP	TRIPC. E3 ubiquitin-protein ligase TRIP12
ASVYA, LEILD	TSC1. Hamartin
ENSVA	TSC2. Tuberin
ALGKL	TUT7. Terminal uridylyltransferase 7
VNLTT	UBP20. Ubiquitin carboxyl-terminal hydrolase 20
LLTDE	UBP28. Ubiquitin carboxyl-terminal hydrolase 28
VNLTT	UBP33. Ubiquitin carboxyl-terminal hydrolase 33
NGTKR, APHGV	UBP7. Ubiquitin carboxyl-terminal hydrolase 7
LQELG	UBXN1. UBX domain-containing protein 1
LPPLL, LQSLQ	UN93A. Protein unc-93 homolog A
DQLTP	USH1C. Harmonin
YKTPP	UVRAG. UV radiation resistance-associated gene protein
GDSSS	VMA5A. von Willebrand factor A domain-containing protein 5A
SNLLL	VRK1. Serine/threonine-protein kinase VRK1
ITPGT	XRN1. 5′-3′ exoribonuclease 1
LAATK	YAP1. Transcriptional coactivator YAP1
AGAAA, CDVVI	ZBT7A. Zinc finger and BTB domain-containing protein 7A
**TAGAAA**, GYLQP, TGTG, STASA, SLQTY	ZFHX3. Zinc finger homeobox protein 3
TQSLL	ZN217. Zinc finger protein 217
SVTTE	ZN276. Zinc finger protein 276
QPELD	ZNRF3. E3 ubiquitin-protein ligase ZNRF3
RSVAS, PDVDL	ZO1. Tight junction protein ZO-1
RSSVL, TSCCS	ZZEF1. Zinc finger ZZ-type and EF-hand domain-containing protein 1

Abbreviations: gp, glycoprotein; SARS-CoV-2, severe acute respiratory syndrome coronavirus 2.
Note: Two-hundred ninety-four proteins have 308 (462, including multiple occurrences) pentapeptides in common with the spike gp antigen. Hexa/heptapeptides deriving from overlapping pentapeptides are given in bold.

 © 2021. The Author(s).
